# How donors support civil society as government accountability advocates: a review of strategies and implications for transition of donor funding in global health

**DOI:** 10.1186/s12992-020-00628-6

**Published:** 2020-11-12

**Authors:** Amy McDonough, Daniela C. Rodríguez

**Affiliations:** Department of International Health, Johns Hopkins Bloomberg School of Public Health, 615 N. Wolfe St., Baltimore, MD 21205 USA

**Keywords:** Donor transitions, Civil society, Advocacy, Accountability, Vulnerable populations

## Abstract

**Background:**

Global health donors are increasingly transitioning funding responsibility to host governments as aid budgets plateau or decline and countries meet development and disease burden goals. Civil society organizations (CSOs) can play a critical role as accountability mechanisms over their governments, but transitions raise questions about how donor-supported CSOs will fare following transition, especially in environments of limited political commitment. Decreases in funding may force CSOs to scale back activities, seek other funding, or rely on their governments for funding. Vulnerable populations most in need of support may lose critical advocates, compromising their access to lifesaving care and threatening the reversal of global health achievements. This review investigates donor strategies used in the past to support CSOs as accountability advocates across the international development sector by exploring what activities are supported, how support is provided and who receives support. It provides considerations for global health donors to better equip civil society as advocates during and following transition.

**Methods:**

A literature review of four databases of peer-reviewed literature, websites focused on civil society support and snowball searching identified 180 documents for review, after application of exclusion criteria, covering up to December 2019. Results were categorized and analyzed by who, what and how donors have supported civil society’s accountability role.

**Results:**

Donors support a variety of civil society actors, including individual organizations and networks, through capacity building, access to information, backing participation in policy dialogues, securing citizen engagement and targeting the broader policy context. Funding may be provided directly or through pooled, intermediary or bridge mechanisms. Key concerns identified include insufficient engagement of CSOs in defining support, limited donor flexibility, tensions in balancing organizational professionalization with community connections, and jeopardized CSO legitimacy and independence from relying on foreign funds.

**Conclusions:**

Given the urgency of global health donor transitions, the literature demonstrates that any donor support to CSO advocates should emphasize transition preparations from the start. Capacity building, institutionalizing mechanisms for civil society participation, planning for information needs, and flexible funding are priority mechanisms to ensure that vulnerable populations continue accessing lifesaving care and global health progress is not reversed.

## Background

Major donors in health, including the Global Fund to Fight AIDS, TB and Malaria (Global Fund), the President’s Emergency Plan for AIDS Relief (PEPFAR), and the Gavi Alliance, are transitioning responsibility for program funding and implementation to national governments. This is due to shifts in the development landscape as well as increases in income status or achievements of disease burden thresholds, and represents a move toward more country ownership of services traditionally funded by donors. Concerns about the ability and commitment of governments to sustain the progress achieved through these health programs abound [1, 2]. These fears are linked to the often substantial support and attention that donors have aimed at services for vulnerable populations, such as sex workers, prisoners or ethnic minorities, in contexts where there is limited political commitment from host governments to serve them. Vulnerable populations are disproportionately burdened by these diseases, but may face marginalization and in some cases criminalization by their own governments [3].

Donors across the international development arena, within and beyond health, have provided substantial financial and technical support to civil society organizations (CSOs) for several decades. Strengthening civil society was seen as a way to increase local participation and efficiency of aid delivery while promoting democratization in the post-Communist era [4]. Support to civil society has risen exponentially since the end of the Cold War brought an emphasis on good governance [5]. Civil society support has since expanded far beyond democracy and governance to other development sectors. Within global health, donors support CSOs both to deliver services, and to advocate for government accountability and rights-based approaches that protect vulnerable populations [1, 3]. This latter advocacy role receives significantly less donor support than service delivery, and when donors withdraw funding, the minimal support for rights and advocacy is often the first to be cut [6], ([7], p. 675), [8].

Donor transitions pose a substantial threat to the role of CSOs as accountability mechanisms. CSOs are likely to face significant funding shortfalls that may force them to scale back their activities, seek other funding sources, or rely on their governments for funding, which may jeopardize their ability to serve as independent advocates [1, 2, 9–11]. Vulnerable populations most in need of support may lose critical advocates, compromising their access to lifesaving care and threatening important global health achievements [3]. Existing challenges within the civil society sector, such as an overreliance on a few professional organizations or inadequate networking across CSOs may be exacerbated by transitions, while new challenges like decreased political space and increased competition for scarce resources may emerge [2].

The area of donor transitions is a growing area of scholarship [12–15], with authors increasingly highlighting the experiences of aid reduction and its impact on civil society [2, 11, 16–19]. However, relatively few pieces have a specific, practical focus on what donors should do to support civil society’s role as advocates and accountability mechanisms amidst donor transitions. There is a greater focus thus far on civil society’s role in service delivery, the need for overall organizational sustainability and issues of accountability to donors as these are fundamental transition-related questions. Given our focus on capturing the strategies available to the donor community to support CSOs as accountability mechanisms post-transition, we have conducted a literature review that allowed us to combine the emerging resources around transitions’ effects on civil society with documentation on CSO support generally to extrapolate what could be done with respect to transitions. The objective is to draw upon the multitude of support strategies that have been used by international development donors within and beyond health to explore *what* specific activities donors may support, *how* to support CSOs with particular funding mechanisms, and *who* to support within civil society. We conclude with considerations for donors to support civil society, whether transition is imminent or far off, in the hopes that civil society can be better prepared and equipped to fulfill its advocacy role during and following transition. Several examples are also included throughout the paper to contextualize the approaches.

### Definition and background of civil society support and advocacy

Civil society can be defined as “all non-market and non-state organisations outside of the family in which people organise themselves to pursue shared interests in the public domain,” including community-based organizations, faith-based organizations, labor unions and other non-governmental organizations (NGOs) ([20], p. 26). For the purposes of this review, the term CSO is used broadly to encompass formal organizations and informal community-based efforts to individual citizen engagement. We broadly define advocacy as participation in any activities with the goal of influencing policy change to improve the wellbeing of underserved populations.

Civil society support comes through a variety of funding mechanisms and donors, including bilateral governments, pooled donor mechanisms and private foundations. External civil society support has raised important debates about what civil society’s role should be and whether donors should even support it in the first place. It has also raised criticisms that the success of civil society strengthening has been limited due to donors’ overly simplistic view of civil society as a homogenous group of organizations that is strengthened merely by increasing the number of organizations, rather than embracing the diversity of this broad group that varies significantly [21], ([22], p. 211). The overall impact of civil society support on political change is a complex question; some find it is unlikely to be a major factor in democratization, and the tendency toward supporting professional NGOs dilutes the political diversity that is often the stated objective of support [23]. While elements of these debates arise in this review, they were beyond the main scope of our exercise. Due to the urgent nature of transitions in which civil society may play a more prominent role, this review seeks to answer *how* to support civil society, rather than *whether* to do so.

## Methods

We conducted a search of peer-reviewed and grey literature that included a wide range of development sectors: agriculture, education, environment, gender, water and sanitation, health, and democracy and governance. The search period went up to and including December 2019.

Key documents [24–27] from the grey literature were used to formulate and verify the search string. The peer-reviewed literature was identified searching Web of Science, PubMed, Embase, and JSTOR using the following search terms:

1. “development,” “donor,” “international,” “foreign”;
2. “assistance,” “aid,” “support,” “funding”;
3. “accountability,” “democracy,” “governance,” “transparency”;
4. “civil society,” “nongovernmental,” “NGO”.

The grey literature was searched to capture more details on the practices used by donors and lessons learned using three methods: (i) search on Google Scholar using the same terms as above; (ii) a snowball approach using key reports, organizational and program documents; and (iii) review of five websites from organizations that either engage directly with or provide resources on support to civil society:

- AIDSPAN’s Global Fund Observer [28];
- The Health Policy Project, supported by the United States Agency for International Development (USAID) [29];
- The Organization for Economic Cooperation and Development’s Development Assistance Committee (OECD-DAC) [30];
- The Open Society Foundations (OSF) [31]; and
- The International NGO Training and Research Centre [32].

Titles and abstracts were screened for the total of 618 peer-reviewed articles and 173 grey literature documents identified. Those included focused on:

- the role that *donors* played in supporting civil society’s role as accountability mechanisms;
- low and/or middle-income countries (LMICs), and former Soviet Union countries that are currently classified as high-income.

Documents were excluded if they:

- focused on civil society advocacy without discussing donor support;
- focused exclusively on civil society’s service delivery role;
- exclusively provided commentary or reflection on the broader role of civil society.

Sixty-five peer-reviewed articles were retained from the database search, and 115 documents from the grey literature search were retained for the review, for a total of 180. Documents were abstracted and analyzed using a literature matrix. Key findings from each document were categorized based on whether they fell into the who, what, or how categories, with the following subcategories for each (Table 1). The matrix also contained categories for general considerations around sustainability, legitimacy and dependency. Following the abstracting process, the authors analyzed key themes within each of the categories, and captured results in this review based on those that were presumed to be of most relevance to transition contexts.

**Table 1:** Categories of literature analysis

Objective category	Subcategories
What	• Capacity building • Support to advocacy activities • Social accountability • Civic education • Access to information • Enabling environment • Rights-based approaches
How	• Bridge funding • Core funding • Pooled funding • Intermediaries • Catalytic funding • General grantmaking considerations
Who	• Networks • Size of organization

## Results

CSOs engage in a wide range of advocacy activities, including direct and formal methods such as advocacy and campaigning or contributing to invited policy dialogue spaces; direct and informal methods such as lobbying or organizing demonstrations; and indirect contributions through disseminating information ([33], p. 62). They engage in invited spaces - where governments formally invite them to participate - and claimed spaces – those which they occupy of their own volition, such as public demonstrations or media engagements ([33], p. 14).

The results are organized by *what* activities donors can support, *how* donors can fund civil society, and *who* within civil society donors can support. They review what has been done and lessons learned, and then are explored within the context of transitions in the Discussion section. We identified a fair amount of variability in nomenclature for methods and modalities of support, which we attempted to streamline, and there is some overlap between sections as the topics are interlinked. The Additional file 1 at the end of the review contains a list of references reviewed.

### What to support

#### Capacity building

Capacity building is an essential, though often overlooked, element of supporting CSOs to engage in advocacy. CSOs require operational capacity to properly function, and technical skills to effectively hold their governments accountable (Table 2). The different types are not mutually exclusive, and providing support to a range of needs is necessary to ensure CSOs are equipped with a full set of skills [33, 36, 45]. In an Embassy-based project of Danish support to civil society, the Embassy provided a range of capacity building, from leadership to monitoring and evaluation, and used a unique “strategic partnership” approach in which it provided ongoing advice to grantees beyond just supporting discrete capacity building activities ([37], p. 58). This type of close engagement is more feasible through embassy support or other instances where the donor is proximate to the recipient. Walker also describes a Gates Foundation-funded program that combined the two types of capacity building for indigenous Nigerian NGOs conducting advocacy around Sustainable Development Goal 3 [34]. Their advocacy resulted in dedicated budget line items for child and family health, and the experience highlighted the importance of strengthening indigenous CSOs, whose voices are often dominated by higher capacity CSOs [34]. The Sustainable HIV Financing in Transition (SHIFT) project, a recent effort to support meaningful involvement of CSOs and key populations in HIV financing in four Global Fund transition countries in Asia, included capacity building targeted toward both CSOs and government officials (see also Table 3) [11].

**Table 2:** Types of capacity building skills

Types of capacity building	Example skills in need of targeting
Operational capacity building: strengthens operating activities of CSO	• Internal monitoring and evaluation systems/skills ([34], p. 59), ([35], p. 5) • Financial management ([36], p. 56), ([37], p. 55), ([38], p. 25), ([39], p. 5) • Program/Project management ([36], p. 50), [34, 39, 40] • Fundraising capacity ([36], p. 50), [31, 38] • Human resource management systems ([34], p. 58) • Leadership skills [39, 41]
Technical capacity building: strengthens specific skills needed to perform effective advocacy	• Budget monitoring skills [36] • Research/data analysis skills ([33], p. 91), [42, 43] • Communications skills [34], ([39], p. 5) • Engaging with the media [44]

**Table 3:** Example of experience with civil society engagement in policy dialogue

The Sustainable HIV Financing in Transition (SHIFT) Project [11]	
The SHIFT project stands out as a recent effort to support meaningful involvement of CSOs and key populations in HIV financing in Indonesia, Malaysia, Thailand and The Philippines. The two-year Global Fund-supported project aimed to equip civil society with the ability and strategic information necessary to advocate for allocative efficiency, increased domestic HIV spending, and increased fiscal space for CSO HIV programs. SHIFT provided grants to larger organizations in each of the countries, who then worked directly with smaller CSOs in their country and established networks and coalitions. Activities varied: capacity building workshops with CSOs and government officials, establishing CSO accreditation systems, supporting the development of a regional knowledge hub to enable learning across CSOs, holding advocacy events, and more ([11], p. 30). While results varied across contexts, the project generated notable policy achievements, such as a national HIV and AIDS Policy Act in the Philippines, and the participation of CSOs in the development of the national HIV Strategy in Indonesia. Twenty-six new official seats were claimed for CSO and key population representatives in domestic funding mechanisms across three of the countries ([11], p. 3). The connections built by the coalitions are expected to live on beyond the project and continue playing an important role in maintaining civil society’s voice and ability to advocate ([11], p. 49). The motivation toward sustainable financing and exposure to government officials is also likely to continue ([11], p. 4).	
While the overall project was deemed successful and replication was recommended, Malaysia saw comparatively less success. The lead grant recipient received most of its funding from the national government, which was viewed by other CSOs in the country as interfering with its independence and ability to openly advocate ([11], p. 24). A key takeaway is that flexibility is needed to properly support advocacy, and donors’ tendencies to request workplans and specific activities at the start of the project negate the very goal of their support ([11], p. 47). Finally, the authors note that as donors transition out, CSOs becoming more reliant on domestic funders makes them no more sustainable because this may jeopardize their independence and ability to advocate, and that sustainability is about “having access to funds that allow CSO to do their work unhindered,” rather than about where the money comes from ([11], p. 6).	

Budget monitoring skills are highlighted as particularly necessary for civil society advocacy ([11], p. 30), ([33], p. 91), [46–48]. The International Budget Partnership supports the use of civil society budget analysis for advocacy, and evidence shows this can impact the budget process across a range of CSOs, while noting that these skills are in short supply [47], ([48], p. 5). Success with budget monitoring has also been seen within the reproductive, maternal and child health field, and calls for strengthening this capacity are echoed. This approach is heavily reliant on budget transparency from governments, which is inadequate in many LMICs ([48], p. 8).

CSOs also require research skills to understand their focus issues and develop effective messages, such as conducting interviews with stakeholders and gathering data, but this is another area often overlooked by donors [33, 49]. Within the context of the Global Fund transition, community representatives voiced a need to strengthen their ability to gather, interpret and use evidence to engage in advocacy and community mobilization, and called for the Global Fund to include them in research processes ([36], p. 38).

While building a diverse skillset is important, targeting multiple levels is also necessary to increase the likelihood of long-term institutional change and combat losses to institutional knowledge from staff turnover [50]. The Health Policy Project (HPP) targeted a combination of individual, organizational, and system-wide levels with government and nongovernmental organizations focused on policy, advocacy and governance (Table 4) ([45], pp. ix, 20). The Advocacy Learning Hubs created by Deutsche Stiftung Weltbevoelkerung (DSW) exemplify an approach to strengthen a variety of skills across different levels for individuals and networks ([48], p. 16). These were formed to build the capacity of East and West African NGOs to fundraise and advocate. Techniques included online and in person training and technical assistance, training individual leaders to train others, and management skills.

**Table 4:** Levels of system to target with capacity building [45]

System Level	Example methods
Individual: strengthens organizational and technical skills of individual advocates	Needs assessments, trainings, distance learning opportunities, technical assistance (e.g. strengthening individual leadership or communication skills)
Organizational: institutionalizes skills and strengthens structures that support organizations	Developing organizational standards and practices (e.g. embedding particular skills into staff job descriptions, strengthening finance systems)
System-wide: supports relationship-building between individuals and organizations	Facilitating cross-learning opportunities and supporting networking (e.g. supporting study visits or online communication and knowledge sharing opportunities)

Experience shows the need to tailor activities to specific needs, and that CSOs must drive the decision about what capacities to build ([33], p. 14), [48, 51, 52]. Past efforts also indicate that building capacity is a long-term endeavor and relies on sustained funding [24, 37, 48]. While trainings are one component, continual support and engagement is needed for well-rounded capacity building that fits into ongoing work with minimal disruption ([11], p. 30), ([48], p. 18), [53]. Pallas and Nguyen also highlight that, while grant writing is a frequently cited option for CSOs to further their own sustainability, the persistent dependence on financial support—regardless of organization size—risks turning the sector more toward donor-driven agendas ([2], p. 146).

Despite the substantial need for capacity building, donors frequently hold high expectations of CSOs to engage in the policy process without addressing capacity gaps to do so ([33], p. 95), [36]. Calls for further capacity building include leadership skills building, articulating clear advocacy asks, using accountability tools, and building CSOs’ institutional governance ([36], p. 50), ([48], p. 17). However, the Consultation on Aid Exits and Locally Led Development (CAELLD), a convening of practitioners involved in donor exits, described capacity building as a useful activity overall, but only when properly executed to account for inherent power dynamics ([17], p. 28). Capacity building can turn into training on meeting donor requirements, which can cause CSOs to adapt to the donor-designed system, and donors tend to work with the easiest to reach, which creates professional intermediaries but limits local engagement ([17], p. 23). A need for local and citizen capacity building, as well as more horizontal and south-south capacity building was expressed ([17], p. 29).

#### Supporting access to data and information

CSOs require data and information about their issue areas to build their understanding and ability to communicate effectively, and donor support for this can range from creating data analysis tools to simply providing internet access ([11], p. 30), [54, 55]. For example, HPP’s Resources for the Awareness of Population Impacts on Development (RAPID) is a free, online advocacy tool used mainly within family planning that uses demographic data to project the consequences of rapid population growth, and helps advocates translate data to policymakers about required family planning resources [54]. CSOs also require tools to navigate advocacy and legislative processes, such as step-by-step guides or toolkits on engaging with the media [56–58].

Providing access to information is an important role for donors, and sometimes the only one they can play in environments where information is limited and governments are skeptical of donor meddling ([59], p. 748). Widening information access can also help level the playing field so a broader set of CSOs can engage [33]. A ‘Resources for All’ approach has been posited, which would encompass publicly available resources, such as information on organizing campaigns or funding opportunities. Increasing access to freely available online resources may help overcome some of the barriers that typically exclude smaller organizations or informal initiatives ([33], p. 111). Information tools can also be combined with networking opportunities between advocates to share knowledge, such as online collaborative learning portals [60]. For example, the SHIFT project created a regional knowledge hub to support networking across CSOs ([11], p. 30).

While information can open doors, caution is warranted around how information access interacts with power dynamics. Supporting larger professional NGOs to access information can widen their divide from smaller, less professional organizations that may have deeper grassroots connections [55].

#### Engaging civil society in policy dialogue

Donors may use their convening power to create space for civil society to engage in or monitor government processes, and they may engage civil society as overseers and participants in their own funding processes (Table 3). Both are, in theory, a way to incorporate the voices of traditionally excluded populations into policy decisions and hold governments and donors accountable. Many authors discuss, however, that merely creating space does not necessarily result in meaningful participation, and that efforts must extend far beyond [61–64]. Donors often support CSOs’ engagement in invited spaces, often a less controversial or politically charged form of engagement, but should also support engagement in claimed spaces, which are often messier but equally important, particularly where governments are not receptive to formal civil society engagement ([33], p. 15).

Several resources describe the risk of tokenistic participation, such as in Eastern Europe where fora to link government and civil society working with people who inject drugs (PWID) left CSOs feeling included in the dialogue for image rather than substance ([61], p. 1751), or in Uganda where the National AIDS Commission used its role in the Global Fund Country Coordinating Mechanism (CCM) to co-opt and consolidate control over civil society [64]. Others point to the Poverty Reduction Strategy Papers process from the World Bank and International Monetary Fund, where ill-defined participation where poor people were invited to meetings, but power dynamics prevented them from talking during meetings and organizers controlled what input was actually taken into account [62], ([65], p. 753).

McNulty highlights that the costs of participation are disproportionately placed on CSOs since they often have to travel and insert themselves into decision-making arenas, which skews toward participation from larger CSOs [63]. Likewise, fitting into donor-designed structures like the CCM may limit CSOs’ ability to fully represent their constituencies and cause them to become a group of actors that cannot challenge the overall system, which limits their empowerment ([62], p. 41), ([66], p. 300). Assessments of Global Fund processes noted that donors need to pay deliberate attention to inequities of engagement, including where dialogue is held, funding travel for those outside urban areas, providing translators at meetings, and ensuring that a representative group is invited and able to participate [23], ([36], p. 34), [67]. A 2017 independent review examined the Global Fund Country Coordinating Mechanisms’ engagement of communities throughout the grant cycle and made a number of recommendations to strengthen this [36].

The OECD-DAC outlines a number of broad characteristics of policy dialogue spaces that support effective CSO participation, such as being regular and systemic, and being designed in a transparent and inclusive manner ([20], p. 61). The important role that donors can play in helping to secure this space was evident in CSOs’ participation in gender-based legislation in Uganda [68].

The adoption of a rights-based approach that includes affected individuals in advocacy and decision-making is cited as crucial [69], ([70], p. 39). As the “nothing about us without us” catchphrase exemplifies, individuals affected by a policy issue are often the best champions [71]. Participation mechanisms need to be designed with the affected populations in mind, and they need to be empowered to engage in decision-making and advocacy ([33], p. 13). Themes throughout the CAELLD’s report reflecting on aid exits and locally-led development highlight the need for more dialogue and inclusion of affected community members and local NGOs from the start of planning ([17], p. 15).

#### Social accountability and citizen empowerment

Social accountability approaches involve “actions other than voting that citizens and civil society can use to hold the state to account…[It enables] communities to assert their political power and to hold local authorities to account” ([72], p. 2). In the health space, these approaches help citizens monitor health service delivery in their communities and hold governments accountable, while strengthening their agency to claim their rights since creating space does not always equal participation ([72], pp. 2), [7, 73]. Tools to build social accountability include community-based data collection and scorecards, health facility surveys, social audits, and budgeting monitoring [74], ([75], p. 6). A rights-based approach that puts vulnerable populations in charge of accountability activities to strengthen their advocacy for their own rights is an essential element of social accountability [74].

Outcomes and impacts of social accountability vary, with both positive and negative results seen [72], ([73], p. 20). Trends identified in contributing to successful social accountability initiatives include adopting flexible and tailored approaches, targeting information to a particular group or issue rather than just providing information, and ensuring that voices are represented to power rather than just expressing voice ([72], p. 6). Consideration must also be given to the negative ramifications that may result from accountability efforts, such as backlash from governments toward accountability advocates [72]. There is also a debate between institutionalizing social accountability mechanisms, or whether emphasizing formality undermines the importance of informal mechanisms, such as community councils, that may build dialogue and trust ([73], p. 18).

#### Targeting the enabling environment and engaging in political analysis

While CSO advocacy can be strengthened via the organizations and individuals engaged in the work, their degree of influence is mediated by the surrounding context, including laws, policies, and regulations that determine the ability of CSOs to engage in development ([20], p. 97), ([50], p. 7). Elements of these preconditions for an effective civil society were laid out in the International Framework for CSO Development Effectiveness, including factors such as freedom of association and legal recognition ([50], p. 7). Donors are criticized for overlooking fundamental elements of the enabling environment and depoliticizing engagement [8], ([33], p. 94), ([50], p. 8), [76]. This depoliticization leads to donors’ overemphasis on providing technical development solutions, rather than assessing and responding to the broader political context in which they seek to spur development [77], which can ultimately stifle a vibrant civil society ([17], p. 24).

Depending on the donor’s relationship with government, it may be able to help improve the institutional environment for CSOs, such as through strengthening governing bodies, exerting political pressure to establish legal rights for CSOs ([33], p. 100) or promoting dialogue at national or international level [40]. Pallas and Nguyen describe the political pressure donors have exerted in Vietnam on behalf of CSOs due to their limited legal standing, and while donors continue to advocate for opening space in light of transition, donors’ reduction of funding may diminish their leverage over the government ([2], p. 139). Within restrictive environments, donors can play a role in defending freedom of expression, assembly and association that are being restrained. In Ethiopia, which has restricted CSOs’ funding and activities, donors pressured the government to relax restrictions and allow more independent engagement, which was noted as contributing to a slight opening of space for CSOs ([78], p. 550). Shared efforts between donors and CSOs to secure democracy and rights in Kenya threaten the government and invite challenges to CSO legitimacy and shrinking space ([79], p. 536). These dynamics are complicated by donors’ desire to maintain strong relationships with government to protect their own interests, causing them to hold back from being fully outspoken in protecting CSO space ([79], pp. 538–9).

Creating legal space is only one piece of the puzzle and needs to be combined with efforts to enable civil society to engage in that space ([33], p. 19), and provide relevant technical assistance that aligns with the local context ([22], p. 199). Political and historical analysis are crucial to defining how to effectively strengthen governance, and for targeting indirect elements of the environment, such as transparent budgeting, better taxation, and policy analysis [76]. A recognition of the power donors have over CSOs, the value that CSOs’ bring to achieving donors’ interests and the role that national efforts play in catalyzing civil society action are strategies to mitigate the occurrence of shrinking civil society space ([79], p. 540).

### How to support

The following section focuses on modalities through which donors may provide support, culminating with general considerations to improve grant-making. The appropriate modality for a country’s transition depends on a careful contextual analysis for both donors and CSOs, so here we review various options for consideration.

#### Core funding

This review identified a repeated call for donors to expand their willingness to provide core support to CSOs, including unrestricted funding, for a range of purposes from supporting administrative operations, like paying salaries and rent, to programmatic work. It contrasts with donors’ tendency to provide restricted project support or employ funding conditionalities, which can dictate CSOs’ agendas and limit their autonomy ([79], p. 539), [81]. Evidence from years of donor support to women’s rights organizations has generated a consensus that core, flexible funding is preferable to project-based funding. It has been seen as vital to strengthen CSOs’ capacity, and saves them time from having to constantly fundraise for core operational support ([38], p. 6) (Table 5). CSOs in the HIV and human rights space echoed the importance of core funding, particularly as funding decreases, and note that insufficient core funding restricts CSOs’ ability to engage effectively in advocacy ([82], p. 7). Core funding can be “crucial and precious” for CSOs as it allows them flexibility, funds to build their organizational capacity to prepare for donor withdrawal, and lessens the potential view that they are beholden to donor agendas ([81], p. 44).

**Table 5:** Mama Cash: Example of pooled funding, core funding, intermediaries, and simplified grantmaking

Mama Cash is a pooled mechanism recognized for providing small, core grants to local advocacy organizations, networks and funds focused on women, girls, trans and intersex groups ([26], p. 6), [80]. The oldest international women’s fund and headquartered in the Netherlands, it supports CSOs with funding for salaries and core operational costs. Mama Cash is praised for its ability to adapt its support to the needs and contexts of individual organizations. Donors that have channeled their money through it include the Dutch Ministry of Foreign Affairs and Irish Aid ([26], p. 6).	
Mama Cash also requires simple application, evaluation and reporting processes to reduce the burden on grantees ([26], p. 6). To capture the impact of its grantees’ advocacy work, at times it uses a participatory tool that helps organizations evaluate behavioral changes that result from their work, rather than emphasizing quantitative indicators ([26], p. 7). Mama Cash is also focusing on providing multi-year funding to reduce the resources grantees must devote to fundraising and enable more long-term planning ([26], p. 7).	

Core funding can contribute to building local ownership by giving recipients greater control over resource allocation ([38], p. 6). It is particularly important for advocacy organizations that need to maintain autonomy and legitimacy given risks of being criticized as having their agendas set by foreign actors ([25], p. 17), [34], ([79], p. 540), [83]. Despite the clear preference for core funding to support their engagement in policy dialogue, CSOs still receive it far less commonly than desired ([33], p. 88), ([79], p. 540). In fact, a recent survey of OECD Development Assistance Committee members found that official development assistance (ODA) flows were going *through* CSOs for project implementation were almost six times higher than *to* CSOs for their operations [40]. HIV/AIDS organizations that have little support for organizational needs, such as salaries and equipment, face a fragile future ([51], p. 1585).

In moving to providing more flexible funding, donors need to be cognizant about defaulting to supporting higher capacity organizations with strong track records at the expense of smaller organizations with limited capacity ([25], p. 17), ([84], p. 20). Additionally, core funding should not be seen as a panacea, and targeted project-based support can be used when donors have a clear understanding of the context and remain cognizant of the need to support the diversity of CSOs ([25], p. 17).

#### Catalytic funding

Catalytic funding can support discrete, short-term, immediate advocacy needs that may not have been initially allocated for. It can quickly support specific activities, rather than invest in a longer term outcome such as organizational capacity strengthening, and can particularly benefit local organizations that may not otherwise receive support ([38], p. 18). Esplen highlights Norway’s Women and Gender Equality Grant, which established catalytic support for gender equality and women’s rights work ([38], p. 18).

The need for catalytic funding aligns with the fact that citizen action is more spontaneous and informal today, in part due to political discontent and the increased availability of electronic communication. This indicates donors need to offer ways to support more fluid forms of organization with flexible, short-term funding ([24], p. 7). This type of funding can, however, lead to high costs for donors since they must be equipped to disburse small batches of funds quickly ([38], p. 18). Given that its strength lies in supporting immediate needs, catalytic funding poses particular relevance to transition environments, where advocacy needs to take place rapidly within uncertain conditions.

#### Donor collaborations/pooled funding

Donors are increasingly drawn to multi-donor collaborations and pooled funding mechanisms, driven in part by the push toward aid effectiveness [24, 84]. Collaborations offer the potential for reduced administrative costs and harmonized support [26], ([37], p. 42) as well as providing greater solidarity in disenabling environments [40]. When agreement across different donors with varying interests is needed, however, this risks working on “lowest common denominator issues” that obtain easy buy-in while neglecting other more complex or politically sensitive issues, and in some cases the best outcomes result from swift and flexible funding ([82], p. 14). This has relevance to vulnerable population advocacy during transition, as donors’ desires to reduce transaction costs risks coming at the expense of supporting more politically sensitive populations.

The UK’s Department for International Development (DfID) has engaged substantially in multi-donor mechanisms to support civil society. Findings from a review of its portfolio include that donors’ transaction costs can decrease when they pool with other like-minded donors, but increase if they need buy-in from other donors to support CSOs in riskier environments or less professionalized organizations. These mechanisms may strengthen local ownership by putting donors more in the backseat, but can also increase the number of donors involved in a CSO’s affairs and inhibit their autonomy [84].

Perspectives on receiving multi-donor funds as a grantee vary. Some CSOs feel it reduces the burden of piecing together funding from different donors and fulfilling many reporting requirements ([82], p. 13). There are concerns among smaller, lesser-known organizations that collaborations fuel a monopolization of resources by more established organizations that already have a relationship with donors ([24], p. 10), ([26], p. 8), ([37], p. 42). Unless funds are deliberately designed to reach smaller organizations, concerns about squeezing out valuable community voices persist ([85], p. 37).

Gaberman, Sovner and Moody reviewed five pooled funds founded after the fall of Communism to support the creation of a vibrant, sustainable civil society and maintain accountability over governments [81]. The funds committed to maintaining a long-term vision, often over 10 years. Some of the organizations initially supported by these funds have grown into successful intermediaries who are able to receive money and ensure it reaches the community level. The pooled fund support laid the foundation for CSOs’ engagement with government while reducing burdensome requirements on organizations from multiple donors and lessening donors’ risk ([81], p. 40).

#### Intermediaries

Intermediary organizations can also be used to disburse funds to local organizations [26]. Types of intermediaries vary widely, including NGOs based in the donor’s country, the country office of an international NGO (INGO), or a country-based NGO ([25], p. 18). Funding through intermediaries offers a way for donors to minimize transaction costs while expanding their reach ([48], p. 26). Pooled intermediaries such as Mama Cash may enable donors to identify organizations with a closer ear to the ground who can support a wider range of local organizations that may not otherwise be reached, including groups that are more politically controversial ([26], p. 5). Investing in larger national organizations also presents a way to develop organizations that can then serve as in-country capacity building resources for smaller organizations [38]. The SHIFT Project demonstrated the potential utility of this approach within the context of transitions [11].

Crowding out may occur when INGO country offices are eligible to apply for the same funding from intermediaries as local CSOs are, and channeling funds through larger organizations to reach smaller ones can create new power dynamics between CSOs ([25], p. 18). Pallas and Nguyen describe how restrictions in Vietnam around funding community-based organizations led donors to rely on a handful of large professionalized intermediaries, leading to concerns that these were profiting middlemen. The authors note this arrangement enhances competition between these intermediaries and stifles networking across civil society due to the lack of incentives to do so ([2], p. 137). The overreliance on intermediaries and the need for greater coalition-building across local NGOs has been noted elsewhere ([17], p. 12).

One strategy to address these dynamics was adopted by the European Commission, which funded CSOs in Bangladesh to work in consortiums, but not to grant to one another ([25], p. 19). Other CSOs note a preference for direct relationships with donors because intermediaries often bring further conditions and demands on funding ([33], p. 88). Donors have also been criticized for rushing to support intermediaries without paying sufficient attention to the accountability and legitimacy of those they choose to support, allowing this to crowd out smaller organizations that contribute to a diverse civil society ([17], p. 12), ([25], p. 23).

Quigley compares the US Government’s use of external intermediaries in Eastern Europe in the 1990s with the European Union’s approach of establishing local foundations to distribute assistance to local NGOs with the country government’s approval. Both came with their own challenges: USAID was hampered by bureaucracy and inflexibility, while the EU’s distribution of smaller grants to many organizations did not generate the strengthened civil society envisioned [22].

While there are risks with intermediaries, experience shows they can be effective if properly selected for the context (Table 6). USAID channeled much of its family planning funding through affiliates of the International Planned Parenthood Federation in the countries it supported, who then distributed funding and technical assistance to local NGOs. Contracting with larger, more capable organizations promoted collaboration and extended support to a more diverse group of organizations than USAID otherwise would have reached. Some of these local organizations lived on beyond USAID’s withdrawal, displaying the value that intermediaries can add ([86], p. 63).

**Table 6:** DfID’s support to the Manusher Jonno Foundation: Example of intermediary funding

Esplen highlights DfID’s Creating Opportunities for Poor and Excluded People (COPE) program in Bangladesh as an example of supporting larger national organizations with closer connections to communities than donors can maintain. The program was implemented by the Manusher Jonno Foundation (MJF), a national NGO, to engage citizens in advocacy and help them exercise their rights. Recognizing MJF’s relationship with local organizations, DfID’s support enabled it to sub-grant to 117 CSOs that work with vulnerable communities ([38], p. 17). These sub-grantees included larger established organizations and smaller organizations that required more support. MJF also provided capacity building support to organizations that had failed to secure MJF’s funding because of insufficient capacity ([38], p. 18). Grantees reported improvements in their management and financial systems, and almost half of the smaller organizations had secured other funding. DfID’s early review of this program identified MJFs’ strengths as its “management capacity…[and] its ability to adapt programmes to local context, activate community support and establish effective links between grassroots work and national policy processes” ([38], p. 18).	
Since these initial findings, MJF was found to have transitioned to funding fewer larger projects in light of high transaction costs during its early years and has been criticized for no longer filling the gap left by short donor-funded projects. It has also come to be viewed as a competitor to its grantees and seen as excluding those with fewer connections, while failing to attract significant further funding ([33], p. 89).	

#### Bridge funding

Bridge funding is one modality specifically proven successful in transition contexts. In preparing for the Global Fund’s transition out of Macedonia, Montenegro and Serbia, OSF provided time-bound bridge grants with other donors to support civil society advocacy [87]. In Macedonia, OSF gave a two-year grant to strengthen the advocacy of a platform of 16 CSOs that advocated for sustainable financing ([87], p. 3). OSF supported the platform to work through the CCM and engage with government, and to organize national protests. A major achievement from this advocacy was that for the first time, the national HIV budget included budget lines addressing the specific needs of different key population groups as opposed to the usual approach of combining all key populations, and the government was planning for social contracting. The government’s allocation to the National HIV Program was four times that of the previous year, and it created legally binding requirements for anti-retroviral therapy support and HIV prevention among key populations ([87], p. 4).

In Montenegro, OSF and the United Nations Development Program (UNDP) supported two CSOs with bridge funding to work with the MOH on developing a social contracting mechanism ([87], p. 7). The government allocated funding for key population services in its call for HIV prevention program proposals, while UNDP funded strengthening components of the legal environment. These examples display how tailored bridge funding can support advocacy and highlights its role in future transition preparations ([87], p. 11).

#### General funding considerations

Regardless of the specific modality used, criticisms abound across sectors of the excessive burden placed on funding recipients by donors [6, 11, 17], ([23], p. 305), ([33], p. 103), [42, 88, 89]. Women’s rights activists, for example, note that CSOs do not lack capacity, they lack capacity to meet donors’ requirements ([38], p. 15). Common complaints highlight overly burdensome grant application and reporting requirements, which create high transaction costs for CSOs and cause them to divert time and resources toward fulfilling donor requirements. High entry barriers create particular challenges for smaller organizations, and often mean larger, professional organizations are more able to access support ([17], p. 23), ([48], p. 18). Private foundations generally have a higher degree of flexibility than government donors who are accountable to taxpayers, and can offer greater flexibility and take more risks with their funding ([23], p. 306). Administrative changes can reduce the burden on CSOs and open doors to lower resource organizations, including relaxing application and reporting requirements, and developing more relevant monitoring and evaluation methods ([26], p. 7), ([33], pp. 15), [21], ([48], p. 19), ([38], p. 14) (Table 5). Some donors’ tendencies to fund short-term, service oriented projects at the expense of advocacy activities that require more long-term support risks depoliticizing civil society and weakening accountability over government. Long-term funding that operates on more realistic timelines can also help mitigate some of these challenges, although this can also fuel dependency [17, 31].

A number of authors discuss the degree to which CSOs need to maintain transparency and links to their grassroots to remain accountable [21], ([22], p. 206), ([33], p. 59), ([50], p. 17), [78]. The receipt of foreign funding risks directing CSOs’ accountability toward their donors while weakening their accountability and connections to their constituencies [9], ([17], p. 14), [79, 90]. Greater efforts to strengthen CSOs’ internal practices, such as inviting community monitoring of their practices or making financial statements public, have been called for to elevate accountability [90, 91]. Accountability from donors downward to civil society and community members is also needed ([17], p. 15).

As dependency on and accountability to foreign donors increase, the potential for criticism around CSO independence and legitimacy heightens [9], ([23], p. 306), ([37], p. 68), ([68], p. 98), [78, 89], as well as the potential for political backlash [9], ([48], p. 17). Receiving donor funding may come with restrictions or strings attached or may turn the recipient’s attention toward donors’ interests rather than their own governments or constituencies. These factors risk weakening their advocacy and inviting questions from skeptical governments [9, 21], ([23], p. 298,300), [37, 78, 91]. Some CSOs completely avoid donor funding because it risks their independence in engaging in policy dialogue ([33], p. 93). Core funding and a diverse donor portfolio are options to mitigate these risks [9, 25, 61, 81].

Sustainability of funding for advocacy CSOs is also a concern [7, 18], ([22], p. 209), [23, 92]. Donor funding has played an essential role in supporting civil society’s work in democracy and rights, and CSOs engaging in this work face the greatest threat from sustainability challenges because they require legitimacy and credibility, regulatory and political space, and financial sustainability [7]. Diversifying the funding base to both international and local sources may also help mitigate this, but the local funding environment is critical [7]. If donor funding ceases and CSOs become reliant on government funding, their ability to openly hold governments accountable may be restricted [2, 9, 10], ([21], p. 21). Alternative funding mechanisms, such as social enterprise, endowments, or crowdfunding are among options to create greater sustainability [2], ([9], p. 10), [81, 93]. Adaptation and flexibility is required from CSOs along with a deep internal reflection about their mission, goals and connections to constituencies, and funders need to seriously consider sustainability in their approaches and build in exit strategies from the start [7]. While there is a persistent focus on long-term sustainability, some efforts may be more oriented toward the short-term, so a reframing of the ultimate goal may need to be rethought ([17], pp. 31–2).

### Who to support

Donors must make complex decisions about who to support and how to balance the tension between creating a diverse civil society with the need to support organizations that can achieve their goals. The following section reviews these debates.

#### Networks/coalitions

Supporting or creating civil society networks can be appealing to reach multiple organizations or actors at once, including lower capacity organizations that may not otherwise be involved (Table 7). Donor assistance can range from supporting established formal networks, to less formal networking activities through individual organizations [11], ([20], p. 121), [81]. Donors may be drawn to funding regional networks, for instance, given their potential for greater capacity, English speaking staff, and ability to draw greater participation ([82], p. 9).

**Table 7:** Robert Carr Civil Society Networks Fund: Example of network support

The Robert Carr Civil Society Networks Fund (RCNF) supports civil society networks working to fight HIV/AIDS. Managed by AIDS Fonds Netherlands, it is a multi-donor mechanism supported by Norad, UK Aid, the Gates Foundation, and PEPFAR. RCNF was the first international fund formed specifically to strengthen international networks around the world. It focuses on those that target the needs and rights of inadequately served populations, and views networks as having the greatest ability to reach populations most affected by HIV. RCNF provides programmatic and core funding to consortia of networks, and global and regional networks [82, 94].	

A benefit of networks is their creation of a collective voice. Coordinated efforts can create a stronger, unified voice and help synchronize resources and enhance knowledge exchange, which can be especially important in restrictive environments to spread risk between organizations ([78], p. 549). Advocates for HIV/AIDS care for PWID found that being part of networks or coalitions strengthened their advocacy and they benefitted from knowledge sharing ([61], p. 1753). Likewise, partnerships between CSO networks in Colombia and Ecuador demonstrate how networks can share experiences and support each other through donor funding fluctuations [89].

However, networks are not easy to create or maintain. Networks can have a narrow focus that creates a “proliferation of uncoordinated networks/coalitions” resulting in rivalry and unhealthy competition within and between them ([61], p. 1753). Other risks to effective networks include fears of engaging with organizations that may be viewed negatively, lack of motivation to pursue shared interests, competition for funding, and inequitable access to resources between urban and rural CSOs ([2], p. 137), ([78], p. 550), ([95], p. 504).

Who represents the constituency, and who they claim to represent, is a critical question ([25], p. 23), [61, 95]. Advocacy networks can be dominated by foreign-funded, service-delivery NGOs, and smaller organizations with less professional expertise may not be included or as able to participate [61, 95]. Networks can become “a network of networks rather than a network of citizens,” with activity mainly occurring between professionals ([95], p. 503).

To create successful networks, efforts include ensuring genuine representation of members with attention to cultural differences and power dynamics, investing in appropriate communications systems, and establishing clarity on incentives for participation ([20], p. 122). Donors should not overly dictate the dynamics since networks take time and effort to manage, and require trust and clarity on the benefits of collaboration ([78], p. 551).

Less formal networking opportunities can also be valuable when the energy required to establish and formalize a network may not be worthwhile, and supporting the process of networking may be more important [33, 76]. For instance, a shared community of practice was created among African civil society Global Fund principal recipients [96].

#### Size and degree of professionalization of CSO

There is debate about whether to support CSOs with greater capacity and proven track records, which tend to be larger traditional NGOs, versus smaller CSOs and movements with lower capacity. A tension exists between balancing a commitment to effectiveness and relying on those who have shown capacity to deliver outcomes, versus supporting diversity and strengthening a strong independent civil society ([24], p. 10). Trends have veered toward supporting the effectiveness side and relying on organizations that already have a proven track record [5], ([17], p. 23), [42, 49, 65, 83]. However, a push toward formalization may actually reduce CSOs’ ability to effectively engage in advocacy and accountability since donors can turn their attention so heavily toward results, rather than the informal networking and long-term change that is needed [55], ([79], p. 539), [89]. Ensuring that support is distributed across a diverse umbrella of civil society requires that, “Donors…be careful not to support the CSOs of today, on the basis of yesterday’s performance at the expense of identifying and supporting tomorrow’s drivers for change” ([24], p. 10). On the other hand, Unsworth notes that in the governance space, donors’ focus at times on creating a diverse civil society spurs a lot of activity that does not end up giving people a voice or impacting policy, fails to acknowledge the power dynamics between civil society and government, and contributes to a proliferation of organizations that are unable to engage with the government [76].

Support to larger organizations does not obviate grassroots connections ([97], p. 394). However, examples display the power dynamics and exclusion that can result from supporting more professional organizations that lack strong grassroots links. In the democracy and governance context, donors have tended to support a small group of professional, urban, wealthy organizations without large memberships or mandates, while excluding smaller grassroots organizations ([5], p. 12). Other experiences echo this tendency toward larger, unrepresentative organizations [55, 83]. While relying on professional NGOs may be administratively easier for donors, other types of groups can play an important role in spurring change, such as in the case of South Africa’s social movement that ended Apartheid ([23], p. 295). Donors are often criticized for idealizing the notion of civil society as a homogenous group of organizations with generally shared interests and goals, without acknowledging the diversity and power inequities encompassed in this very large space ([4], p. 3), [17], ([22], p. 211), ([50], p. 15), [76].

To address the tension regarding organization size and track record, several options have been highlighted. One is to build the capacities of larger national organizations with links to their constituencies so they can strengthen local organizations moving forward [38], which gives intermediaries the opportunity to invest in larger organizations that can support smaller local organizations ([48], p. 26). The SHIFT project demonstrates a largely successful use of this approach within the transition context [11]. Another option is for donors to provide smaller amounts of funding to multiple small organizations at once ([48], pp. 16, example 4.2.1). Support to individuals who can ultimately lead organizations can also be important [81].

In certain contexts, where a strong foundation of civil society does not exist, supporting a smaller group of organizations to ensure there is a support base for civil society may be the most effective use of scarce resources. In the European Union’s support to Kosovo, the most urgent need was not to create a pluralistic, vibrant civil society, but rather to establish a critical base of organizations with the capacity to accomplish policy reform goals [98]. Donors must understand the current structure of civil society within a given country, and then decide how to intervene ([23], p. 296).

Beyond focusing on support to organizations, individual empowerment is cited as a missing link in holding government accountable, and civic education and empowerment is also necessary for long term change ([17], p. 27), ([49], p. 52). Donor funding to NGOs to support citizen empowerment initiatives can skew organizations’ accountability toward donors, and these more professional organizations cannot always claim to lead or represent grassroots interests [95]. Chahim and Prakash quote Pearce’s assertion that, “constructing civil society cannot be essentially about building up intermediary development organizations to represent the ‘poor’; it must be about empowering the poor and enabling them to fight for their own rights as citizens” ([95], p. 510), ([99], p. 225).

## Discussion

This review has described the ways in which donors support civil society, including what activities they support, which organizations they target and how they are funded (Table 8). Types of support to CSOs include capacity building, engagement in policy dialogue, improving access to information, and influencing the enabling environment. The review uncovers a desire from CSOs to be more meaningfully involved in determining what activities should be supported.

**Table 8:** Summary of ways in which donors support CSOs and general themes

Category of support	Summary of ways in which donors support CSOs
What	• Donors may support CSOs and individuals with technical and operational capacity building, or provide them with access to information to increase awareness and analysis capacity. • Donors may work to ensure civil society is equipped to meaningfully participate in policy dialogue. • Building social accountability and citizen empowerment is a strategy to equip citizens with the tools to exert oversight over decisionmakers. • Donors may use their political capital to target the broader enabling environment in which CSOs operate.
How	• Donors may provide catalytic of bridge funding to help CSOs address specific needs or seize an opportune moment. • Funding may be provided through intermediaries to extend the reach of donor organizations, donors may participate in pooled funding mechanisms, or funding may be provided directly to CSOs. • Core funding is seen as vital by CSOs, but is given less frequently than restricted funding. • Burdensome reporting or application requirements are examples of improvements needed in donor funding practices called for by CSOs.
Who	• Tensions exist around whether to support large established organizations versus smaller organizations, raising questions of whether those representing certain constituencies have a strong connection to those they represent. • Donors may also support networks to reach a larger number of organizations at once.

In terms of organizations, small CSOs, large professional NGOs, and regional networks are all recipients of donor support via a variety of mechanisms, including core, catalytic, pooled and bridge funding, and intermediaries. There are unresolved tensions on whether donor support should target organizations already professionalized or focus on ensuring strong ties to constituencies. This is intricately linked with questions of capacity to manage foreign aid and meet grant reporting requirements as well as risks to independence and legitimacy, especially in politically charged environments. In terms of capacity and legitimacy, research in Bosnia-Herzegovina highlights how CSOs who function as successful intermediaries between citizens and policymakers require legitimacy in both camps and two types of capacity to succeed: transactional capacity to engage politicians and participatory capacity to engage citizens [100].

The review also highlights a critical ingredient for successful support to civil society: donors need to understand the local policy environment and the universe of civil society actors in order to successfully support CSOs as an accountability mechanism for services. Civil society engagement in policy dialogue cannot be tokenistic and requires CSOs to have the agency and ability to meaningfully engage. Supporting efforts that influence the broader context to promote a more functional, democratic enabling environment are critical. Further, not only should donors pursue a robust, varied portfolio of CSOs, likewise CSOs should be encouraged to consider their own sustainability strategies within the context of their short and long-term goals.

### Considerations for transition

Despite civil society’s critical role in demanding accountability for vulnerable populations, relatively little of the reviewed literature directly addresses the implications and practical considerations regarding donor transition for civil society to play this advocacy and accountability role, so here we draw out a few relevant ideas for consideration based on research evaluating donor transitions [2, 11–13, 15, 17, 81, 101]. The review shows that civil society is best supported through long-term engagement, so preparing for transition and an exit strategy needs to be well-planned and even considered when initiating support to a CSO [2, 7, 16, 17, 102]. Where possible, strategies that address multiple parts of the system simultaneously pose the greatest likelihood for success. Strengthening legal space for civil society, for instance, may have minimal impact if those legal frameworks are not then enacted. Successful transition examples were marked by close consultation and open communication with CSOs in the transition planning process ([17], p. 36). A continued threat is misaligned interests between donors eager to see their service provision continued, and CSOs eager to sustain an independent civil society and maintain the welfare of their members ([2], p. 143).

In assessing *what* to support, capacity building is essential [103]. CSOs will face difficulties sustaining efforts if they are not equipped with the basic skills required to conduct advocacy. If leadership or communication skills are lacking, or individuals do not have adequate training in research or budget monitoring, for instance, they may be less able to assert themselves in policy fora and at risk of tokenistic inclusion, a risk which will be exacerbated as donors leave. Capacity building should be designed in partnership with civil society and must account for inherent power dynamics, while considering both short-term and long-term goals of advocacy or accountability efforts ([17], p. 28).

Donors can also use their convening power while they are still present to help institutionalize the methods of engagement so they continue once they leave, such as the Community Taskforces the Global Fund has helped institute or USAID’s legacy planning for NGO support in Croatia [103]. Donors must consider power inequities between actors and embed a rights-based approach into participation mechanisms. The degree to which vulnerable populations are publicly engaged in advocacy may depend on their levels of safety and comfort in engaging directly with government or in other public fora, but their inclusion in behind the scenes decision-making is no less crucial. The populations most at risk of being impacted by transition may be the most politically sensitive, so extra attention is required to ensure they are not neglected.

Access to information may be threatened following transition, so CSOs’ ability to gather evidence, communicate with other CSOs, and access tools that help guide them through the advocacy process may be lost. Supporting knowledge sharing between CSOs, such as through online platforms, presents a way to harness the knowledge that lies within civil society and help them support one another outside of donor support. In place of donor technical assistance, tools to guide CSOs through the policy process, like those developed by HPP, present an opportunity to provide lasting resources to guide CSOs [54].

Funding through intermediaries during and after transition appears to present the greater opportunity where the goal is to reach a larger set of local organizations, either to drum up broad pressure over government, or to strengthen their advocacy capacity [11]. Where this capacity does not exist, donors could strengthen the capacity of one or a few intermediaries so they can work with local CSOs following transition or promote South-South development cooperation among CSOs [89]. However, where needs are urgent and an organization with a proven track record is needed to quickly engage in advocacy, intermediaries are probably not the answer. While domestic government funding is one way that civil society may sustain following donor withdrawal, governments’ willingness to fund CSOs cannot be assumed and it may risk their independence and legitimacy. Donors may also consider supporting further financial sustainability efforts, such the development of local philanthropy or helping CSOs start social enterprises ([2], p. 146), ([11], p. 6), [18].

The findings regarding *who* to support are heavily dependent on the context. Where time is short and advocacy needs are immediate, it seems most realistic to focus on supporting key organizations or networks with targeted support, such as OSF’s support in Macedonia, to ensure that critical advocacy is supported during the pre-transition window. Building up a select few groups within civil society to ensure there is a foundational base to carry forth advocacy following donor exit may be the most impactful option [11]. Where transition is less imminent, donors may work through intermediaries or provide smaller grants to strengthen a broader array of organizations and look to support more informal initiatives and movements. Donors need to carefully consider what the goal of support is – such as whether to secure specific advocacy wins, or to build up a diverse, large civil society.

We have synthesized a list of good practice lessons for donors facing transition depending on the available time horizon, taking into account how donors’ power and influence can be catalyzed before a transition (Table 9). While many of the reviewed works were from non-transition contexts, the strategies proposed – such as building an exit strategy from the start, ensuring meaningful inclusion of those affected, and building capacity to sustain following withdrawal – apply across a variety of contexts. While an impending transition may add urgency and change some dynamics of the donor-CSO relationship, the review highlights that these practices should be undertaken at any time of engagement, even when transition is far off. Additional analysis is needed as more transition-specific examples emerge.

**Table 9:** Good practices for donors facing transition

Transition timing	Approaches
Impending transition	➢ **Strengthen key organizations and individuals** who can carry advocacy forward following transition, and who present the potential to serve as country-level and future capacity building resources for smaller organizations. ➢ **Support networks** with robust existing capacity to mobilize advocacy, or encourage the unification of CSOs, while ensuring community needs are represented. ➢ **Channel funding through intermediaries** to create broad advocacy support and pressure over governments. ➢ **Create meaningful participatory mechanisms** to bring together civil society and government to agree to rules of engagement and codify participation. ➢ **Provide time-bound, flexible bridge grants,** with catalytic funding available to support targeted advocacy.
Longer-term engagement	➢ **Diversify funding modalities** to support a mix of organizations, potentially through donor coordination mechanisms and intermediaries. ➢ **Support longer-term capacity building** across different levels of the system – individual, organizational, and systemic - and define in tandem with CSOs what support is needed. ➢ **Support social accountability efforts** that engage members of affected populations to generate public engagement and demand and hold their government to account.
Changing donors’ approach at any point of engagement	➢ **Provide more flexible funding, reporting and evaluation** with core support, looser grant application and reporting requirements, and improved monitoring and evaluation to better measure advocacy. ➢ **Build an exit strategy and consider sustainability from the start** in partnership with CSOs ➢ **Support rights-based activities**, particularly organizations and activities that include or are led by members of affected populations, and focus on embracing the diversity of vulnerable populations. ➢ **Address enabling environments** by recognizing the historical and political context in which civil society sits, and adjust donor involvement accordingly. ➢ **Strengthen access to data and information** via knowledge sharing hubs and online tool repositories. ➢ **Consider non-traditional modes of engagement** like informal initiatives and mobilization via social media.

### Limitations

There are several limitations to this review. First, regarding our focus and search approach. “Donor transition” is an emerging area of scholarship so resources directly dealing with civil society support around transition, especially as it relates to CSOs in a government accountability role are limited. By focusing our inclusion/exclusion process on the title and abstract, we may have missed publications that discuss accountability and advocacy within the main text. Second, other activities or modalities with relevance to transition that receive substantial donor support, such as civic education, were not thoroughly addressed in this review. We also did not assess how donors should support CSOs’ service delivery roles amidst transitions as it was outside the scope of this review. There are also inherent relational differences between grantees and donors that were not fully addressed in the review. For instance, bilateral donors who need to maintain a diplomatic relationship with the host government may operate differently than a private foundation that fully exits following transition. All of these are opportunities for further analysis.

Third, the literature reviewed ended up being primarily focused on the health sector. This may be because (i) one of the peer-reviewed databases used is specifically health-focused, and (ii) the significant donor funding and attention to health, and particularly HIV/AIDS, has generated a large amount of literature around civil society advocacy, which may have driven the large number of health-specific results. Likewise, two of the organizations used in the grey literature search are health-focused likely reflecting the researchers’ professional background in health.

Lastly, we were unable to assess the effectiveness of the support strategies discussed. Evaluating the impact of efforts to influence policy change and hold governments accountable is inherently difficult and identifying the direct impact of donor support even more so. While an effort was made to tease out effectiveness information where possible, this review highlights anecdotal discussions of effectiveness only. Further research to measure effectiveness is needed.

## Conclusion

There is no one-size-fits-all approach for donor support to civil society, and support is still not being sufficiently contextualized. Transitions will only exacerbate the need for this, as only support that is tailored to the specific environment will stand a chance of lasting effects after exit. To adequately prepare, donors must work with civil society to assess the specific contextual needs, including the time horizon available for support, the political environment in which civil society operates, and donors’ relationships with civil society and governments.

The findings from decades of civil society support display myriad ways to support civil society’s role as accountability advocates, and donor transitions will elevate the importance of this role played by civil society. Donors must assess their own positions and identify where they will be most impactful, while being cognizant of their impact on the legitimacy, accountability and sustainability of CSOs. As donor transitions continue, support to strengthen CSOs as advocates is urgently needed to ensure that key global health achievements are not reversed and vulnerable populations do not lose access to lifesaving services.

## Supplementary information

**Additional file 1.** Other Works Reviewed.

## Data Availability

Not applicable.

## Abbreviations

AIDS: Acquired immunodeficiency syndrome
CCM: Country Coordinating Mechanism
CAELLD: Consultation on Aid Exits and Locally Led Development
CSO: Civil society organization
DfID: Department for International Development
HIV: Human immunodeficiency virus
HPP: Health Policy Project
INGO: International nongovernmental organization
INTRAC: International NGO Training and Research Centre
LMICs: Low and/or middle-income countries
MOH: Ministry of Health
NGO: Nongovernmental organization
OECD-DAC: Organisation for Economic Development-Development Assistance Cooperation
ODA: Official development assistance
OSF: Open Society Foundations
PEPFAR: President’s Emergency Plan for AIDS Relief
PWID: People who inject drugs
RAPID: Resources for the Awareness of Population Impacts on Development
SHIFT: Sustainable HIV Financing in Transition project
UNDP: United Nations Development Program
USAID: United States Agency for International Development
