# Supplementary material for: How donors support civil society as government accountability advocates: a review of strategies and implications for transition of donor funding in global health
Source: Global Health. 2020 Nov 12;16:110. doi: 10.1186/s12992-020-00628-6 (PMC7659168; doi:10.1186/s12992-020-00628-6)
Supplement: Supplementary file 1 — Additional file 1. Other Works Reviewed. [file 12992_2020_628_MOESM1_ESM.docx]

**Additional file 1: Other Works Reviewed**

Aidspan Staff, “Global Fund issues RFPs on community engagement,” *AIDSPAN*, Jun. 13, 2017. <http://www.aidspan.org/gfo_article/global-fund-issues-rfps-community-engagement>.

T. Abdel-Malek, F. Leautier, and F. Straface, “Capacity Development - Lessons learned from Busan: Synthesis Report,” High Level Group on Capacity Development, Cairo, Egypt, Mar. 2011. [Online]. Available: <http://www.oecd.org/dac/accountable-effective-institutions/48146228.pdf>.

C. AbouZahr *et al.*, “Civil registration and vital statistics: progress in the data revolution for counting and accountability,” *The Lancet*, vol. 386, no. 10001, pp. 1373–1385, Oct. 2015, doi: [10.1016/S0140-6736(15)60173-8](https://doi.org/10.1016/S0140-6736(15)60173-8).

S. Appe, “NGO Networks, the Diffusion and Adaptation of NGO Managerialism, and NGO Legitimacy in Latin America,” *Voluntas: International Journal of Voluntary & Nonprofit Organizations*, vol. 27, no. 1, pp. 187–208, Feb. 2016, doi: [10.1007/s11266-015-9594-y](https://doi.org/10.1007/s11266-015-9594-y).

E. L. Aveling, “The impact of aid chains: relations of dependence or supportive partnerships for community-led responses to HIV/AIDS?,” *AIDS Care*, vol. 22, no. sup2, pp. 1588–1597, Dec. 2010, doi: [10.1080/09540121.2010.507954](https://doi.org/10.1080/09540121.2010.507954).

G. Baiocchi, P. Heller, and M. K. Silva, “Making Space for Civil Society: Institutional Reforms and Local Democracy in Brazil,” *Social Forces*, vol. 86, no. 3, pp. 911–936, 2008, [Online]. Available: <http://www.jstor.org/stable/20430782>.

C. Baran, “Key population networks call for strengthening engagement in grant-making and implementation,” *AIDSPAN*, Apr. 03, 2017. <http://www.aidspan.org/gfo_article/key-population-networks-call-strengthening-engagement-grant-making-and-implementation>.

C. Baran, “Six Regional Platforms for Communication and Coordination begin work as part of the Global Fund’s CRG Strategic Initiative | Aidspan,” *AIDSPAN*, Nov. 28, 2017. <http://www.aidspan.org/gfo_article/six-regional-platforms-communication-and-coordination-begin-work-part-global-fund%E2%80%99s-crg>.

D. M. C. Bartlett, “Civil Society and Democracy: A Zambian Case Study,” *Journal of Southern African Studies*, vol. 26, no. 3, pp. 429–446, 2000, [Online]. Available: <http://www.jstor.org/stable/2637411>.

S. Bennett, A. Corluka, J. Doherty, and V. Tangcharoensathien, “Approaches to developing the capacity of health policy analysis institutes: a comparative case study,” *Health Research Policy and Systems*, vol. 10, p. 7, Mar. 2012, doi: [10.1186/1478-4505-10-7](https://doi.org/10.1186/1478-4505-10-7).

C. Bodewes, “Civil society and the consolidation of democracy in Kenya: an analysis of a atholic parish’s efforts in Kibera slum,” *The Journal of Modern African Studies*, vol. 48, no. 4, pp. 547–571, 2010, [Online]. Available: <http://www.jstor.org/stable/40961828>.

D. S. Brown, J. C. Brown, and S. W. Desposato, “Promoting and Preventing Political Change through Internationally Funded NGO Activity,” *Latin American Research Review*, vol. 42, no. 1, pp. 126–138, 2007, [Online]. Available: <http://www.jstor.org/stable/4499356>.

G. Cameron, “Background paper: How should we classify civil society? A review of mainstream and alternative approaches,” INTRAC, Oxford, UK, 2008. [Online]. Available: <https://www.sociedadenaccion.cl/wp-content/uploads/2015/06/Cameron-G.-2008-Background-paper.-How-should-we-classify-civil-society.-A-review-of-mainstream-and-alternative-approaches.pdf>.

J. Carmin, “NGO capacity and environmental governance in Central and Eastern Europe,” *Acta Politica; Basingstoke*, vol. 45, no. 1–2, pp. 183–202, Apr. 2010, doi: <http://dx.doi.org/10.1057/ap.2009.21>.

C. Collins, T. Coates, and G. Szekeres, “Accountability in the Global Response to HIV,” *AIDS*, vol. 22, no. Suppl 2, pp. S105–S111, Aug. 2008, doi: [10.1097/01.aids.0000327442.66656.01](https://doi.org/10.1097/01.aids.0000327442.66656.01).

J. Crotty, “Managing civil society: democratisation and the environmental movement in a Russian region,” *Communist and Post-Communist Studies*, vol. 36, no. 4, pp. 489–508, Dec. 2003, doi: [10.1016/j.postcomstud.2003.09.006](https://doi.org/10.1016/j.postcomstud.2003.09.006).

M. Daku, “Activists urge continuation of special investments in community, rights and gender,” *AIDSPAN*, Nov. 01, 2016. <http://www.aidspan.org/gfo_article/activists-urge-continuation-special-investments-community-rights-and-gender>.

E. L. Dawson, “Gender, diversity, and sustainable civil society strengthening: lessons from Ethiopia,” *Development in Practice*, vol. 26, no. 5, pp. 629–636, Jul. 2016, doi: [10.1080/09614524.2016.1190317](https://doi.org/10.1080/09614524.2016.1190317).

S. Devarajan, S. Khemani, and M. Walton, “Can Civil Society Overcome Government Failure in Africa?,” *World Bank Research Observer*, vol. 29, no. 1, pp. 20–47, Feb. 2014, [Online]. Available: <http://search.ebscohost.com/login.aspx?direct=true&db=eoh&AN=1432957&site=ehost-live&scope=site>.

J. L. Doyle, “Civil Society as Ideology in the Middle East: A Critical Perspective,” *British Journal of Middle Eastern Studies*, vol. 43, no. 3, pp. 403–422, Jul. 2016, doi: [10.1080/13530194.2015.1102713](https://doi.org/10.1080/13530194.2015.1102713).

O. G. Encarnación, “Tocqueville’s Missionaries: Civil Society Advocacy and the Promotion of Democracy,” *World Policy Journal*, vol. 17, no. 1, pp. 9–18, 2000, [Online]. Available: <http://www.jstor.org/stable/40209672>.

S. E. Finkel, C. A. Sabatini, and G. G. Bevis, “Civic Education, Civil Society, and Political Mistrust in a Developing Democracy: The Case of the Dominican Republic,” *World Development*, vol. 28, no. 11, pp. 1851–1874, Nov. 2000, doi: [10.1016/S0305-750X(00)00067-X](https://doi.org/10.1016/S0305-750X(00)00067-X).

J. Fisher, “Local and Global: International Governance and Civil Society,” *Journal of International Affairs*, vol. 57, no. 1, pp. 19–39, 2003, [Online]. Available: <http://www.jstor.org/stable/24357911>.

A. Fowler, “Non-governmental development organisations’ sustainability, partnership, and resourcing: futuristic reflections on a problematic trialogue,” *Development in Practice*, vol. 26, no. 5, pp. 569–579, Jul. 2016, doi: [10.1080/09614524.2016.1188883](https://doi.org/10.1080/09614524.2016.1188883).

D. Garmaise, “Global Fund releases case studies on community engagement,” *AIDSPAN*, Feb. 27, 2017. <http://www.aidspan.org/gfo_article/global-fund-releases-case-studies-community-engagement>.

S. Godt, S. Mhatre, and A.-M. Schryer-Roy, “The change-makers of West Africa,” *Health Research Policy and Systems*, vol. 15, no. 1, p. 52, Jul. 2017, doi: [10.1186/s12961-017-0208-6](https://doi.org/10.1186/s12961-017-0208-6).

P. Grajzl and P. Murrell, “Fostering Civil Society to Build Institutions: Why and When,” *Economics of Transition*, vol. 17, no. 1, pp. 1–41, 2009, doi: [10.1111/%28ISSN%291468-0351/issues](https://doi.org/10.1111/%28ISSN%291468-0351/issues).

A. Hadenius and F. Uggla, “Making civil society work, promoting democratic development: What can states and donors do?,” *World Development*, vol. 24, no. 10, pp. 1621–1639, Oct. 1996, doi: [10.1016/0305-750X(96)00062-9](https://doi.org/10.1016/0305-750X(96)00062-9).

C. Halmshaw and K. Hawkins, “Capitalising on Global HIV/AIDS Funding: The Challenge for Civil Society and Government,” *Reproductive Health Matters*, vol. 12, no. 24, pp. 35–41, 2004, [Online]. Available: <http://www.jstor.org/stable/3776580>.

K. A. Hartwig, D. Humphries, and Z. Matebeni, “Building capacity for AIDS NGOs in southern Africa: evaluation of a pilot initiative,” *Health Promotion International*, vol. 23, no. 3, pp. 251–259, doi: [10.1093/heapro/dan013](https://doi.org/10.1093/heapro/dan013).

J. Hearn, “Aiding Democracy? Donors and Civil Society in South Africa,” *Third World Quarterly*, vol. 21, no. 5, pp. 815–830, 2000, [Online]. Available: <http://www.jstor.org/stable/3993620>.

J. Hedman and I. McDonnell, “How DAC members work with civil society,” OECD, 2011. [Online]. Available: <https://www.oecd.org/dac/peer-reviews/Final_How_DAC_members_work_with_CSOs%20ENGLISH.pdf>.

E. Howe, “Bulgaria’s Roma Pushing for Real Accountability in Health Care,” *Open Society Foundations*, Aug. 22, 2012. <https://www.opensocietyfoundations.org/voices/bulgaria-s-roma-pushing-real-accountability-health-care>.

A. Ithibu, “New fund to be launched to support meaningful engagement of adolescent girls and young women in Global Fund and national processes,” *AIDSPAN*, Nov. 07, 2017. <http://www.aidspan.org/gfo_article/new-fund-be-launched-support-meaningful-engagement-adolescent-girls-and-young-women>.

M. A. Jamal, “Democracy Promotion, Civil Society Building, and the Primacy of Politics,” *Comparative Political Studies*, vol. 45, no. 1, pp. 3–31, Jan. 2012, doi: [10.1177/0010414010365998](https://doi.org/10.1177/0010414010365998).

A. Jorgenson, “‘I’m Already Changed’: Women’s Leadership Workshop Acts as Catalyst for Change,” Jul. 14, 2014. <http://www.healthpolicyproject.com/index.cfm?id=WomensLeadership2014>.

S. Lewis, A.-A. Boateng, and R. Hayman, “Building sustainability of civil society: Debates, challenges and moving forward,” INTRAC, Oxford, UK, Jan. 2015. [Online]. Available: <https://www.intrac.org/wpcms/wp-content/uploads/2016/09/Debates-challenges-and-moving-forward.-Lewis-Boateng-Hayaman.-INTRAC-2015.pdf>.

K. Lotshwao, R. Imre, and J. Jose, “Democracy Assistance for Botswana: Maintaining the Status Quo in a Peripheral Capitalist Country,” *Journal of Developing Societies*, vol. 35, no. 2, pp. 205–229, Jun. 2019, doi: [10.1177/0169796X19843361](https://doi.org/10.1177/0169796X19843361).

O. Lutsevych, “Civil society versus captured state: a winning strategy for sustainable change,” *Development in Practice*, vol. 26, no. 5, pp. 646–656, Jul. 2016, doi: [10.1080/09614524.2016.1188885](https://doi.org/10.1080/09614524.2016.1188885).

R. S. Magnusson and D. Patterson, “The role of law and governance reform in the global response to non-communicable diseases,” *Globalization and Health*, vol. 10, p. 44, Jun. 2014, doi: [10.1186/1744-8603-10-44](https://doi.org/10.1186/1744-8603-10-44).

L. Messerschmidt, “Thematic Study 2: Taskforces: Building Resilient and Collaborative Systems for Health Advocacy,” Community Action and Leadership Collaborative, Dec. 2016. [Online]. Available: <http://msmgf.org/wp-content/uploads/2017/03/Building-Resilient-and-Collaborative-Systems-for-Health-Advocacy-2017.pdf>.

L. Messerschmidt, “Thematic Study 1: Engagement in Governance and Decision-making Structures,” Community Action and Leadership Collaborative, Jan. 2017. [Online]. Available: <https://msmgf.org/wp-content/uploads/2017/03/Engagement-in-Governance-and-Decision-Making-Structures-2017.pdf>.

G. Mohan, “The disappointments of civil society: the politics of NGO intervention in northern Ghana,” *Political Geography*, vol. 21, no. 1, pp. 125–154, Jan. 2002, doi: [10.1016/S0962-6298(01)00072-5](https://doi.org/10.1016/S0962-6298(01)00072-5).

R. Nandelenga, “Demanding a high impact HIV response: Civil society advocacy and the President’s Emergency Plan for Aids Relief (PEPFAR) in Uganda,” *J. Int. AIDS Soc.*, vol. 19, no. 6 (Suppl 5), 2016, doi: [10.7448/IAS.19.6.21264](https://doi.org/10.7448/IAS.19.6.21264).

T. O’Neill, M. Foresti, and A. Hudson, “Evaluation of Citizens’ Voice & Accountability: Review of the Literature & Donor Approaches Report,” DFID, London, 2007.

G. Oberth, “Assessing the Inclusion of Civil Society Priorities in Global Fund Concept Notes,” Eastern Africa National Networks of AIDS Service Organization, Arusha, Tanzania, Aug. 2015. [Online]. Available: <http://www.globalfundadvocatesnetwork.org/wp-content/uploads/2015/10/EANNASO-2015-Assessing-the-Inclusion-of-Civil-Society-Priorities-in-Global-Fund-Concept-Notes.pdf>.

G. Oberth, “Report on regional grants finds information barriers for civil society | Aidspan,” *AIDSPAN*, Sep. 29, 2016. <http://www.aidspan.org/gfo_article/report-regional-grants-finds-information-barriers-civil-society>.

G. Oberth, “Shadow reports on CCM performance flag issues of oversight and conflict of interest | Aidspan,” *AIDSPAN*, Mar. 07, 2017. <http://www.aidspan.org/gfo_article/shadow-reports-ccm-performance-flag-issues-oversight-and-conflict-interest>.

G. Oberth, “Board approves $15 million for continuation of strategic investments in community, rights and gender over 2017-2019,” *AIDSPAN*. <http://www.aidspan.org/gfo_article/board-approves-15-million-continuation-strategic-investments-community-rights-and-gender>.

G. Oberth and M. Daku, “Global Fund tops PEPFAR on engagement of key populations: Survey,” *AIDSPAN*, Aug. 10, 2016. <http://www.aidspan.org/gfo_article/global-fund-tops-pepfar-engagement-key-populations-survey>.

OECD/UNDP, “Making Development Co-operation More Effective: 2016 Progress Report,” OECD Publishing, Paris, 2016. [Online]. Available: <http://dx.doi.org/10.1787/9789264266261-en>.

M. Orozco and L. G. P. Farina, “HIV/AIDS Policy in Nicaragua: A Civil Society Perspective,” Open Society Institute, 2007. [Online]. Available: <https://www.opensocietyfoundations.org/sites/default/files/nicaragua_20080115.pdf>.

A. D. Oxman, S. Lewin, J. N. Lavis, and A. Fretheim, “SUPPORT Tools for evidence-informed health Policymaking (STP) 15: Engaging the public in evidence-informed policymaking,” *Health Research Policy and Systems*, vol. 7, no. 1, p. S15, Dec. 2009, doi: [10.1186/1478-4505-7-S1-S15](https://doi.org/10.1186/1478-4505-7-S1-S15).

I. M. Pousadela and A. Cruz, “The sustainability of Latin American CSOs: historical patterns and new funding sources,” *Development in Practice*, vol. 26, no. 5, pp. 606–618, Jul. 2016, doi: [10.1080/09614524.2016.1188884](https://doi.org/10.1080/09614524.2016.1188884).

X. Rambla, A. Verger, D. B. E. Jr, C. Fontdevila, and X. Bonal, “Meeting development goals: evidence from the Civil Society Education Fund,” *Development in Practice*, vol. 27, no. 6, pp. 851–864, Aug. 2017, doi: [10.1080/09614524.2017.1343275](https://doi.org/10.1080/09614524.2017.1343275).

A. P. Singh, “Civil Society and Good Governance: Emerging Challenges,” *Journal of the Indian Law Institute*, vol. 50, no. 1, pp. 81–93, 2008, [Online]. Available: <http://www.jstor.org/stable/43952134>.

J. Smith, K. Buse, and C. Gordon, “Civil society: the catalyst for ensuring health in the age of sustainable development,” *Globalization and Health*, vol. 12, no. 1, p. 40, Jul. 2016, doi: [10.1186/s12992-016-0178-4](https://doi.org/10.1186/s12992-016-0178-4).

N. Spina and C. Raymond, “Civil Society Aid to Post-communist Countries,” *Political Studies*, vol. 62, no. 4, pp. 878–894, Dec. 2014, doi: [10.1111/1467-9248.12081](https://doi.org/10.1111/1467-9248.12081).

J. Ssozi, “In Uganda, Grassroots Radio Bridges Health Care Gaps,” *Open Society Foundations*, Jul. 09, 2013. <https://www.opensocietyfoundations.org/voices/uganda-grassroots-radio-bridges-health-care-gaps>.

M. Strecker, M. Stuttaford, and L. London, “Health rights pamphlets: critical literacy and inclusive citizenship, South Africa,” *Health Promotion International*, vol. 29, no. 2, pp. 339–348, Jun. 2014, doi: [10.1093/heapro/das067](https://doi.org/10.1093/heapro/das067).

L. M. Sundstrom, “Foreign Assistance, International Norms, and NGO Development: Lessons from the Russian Campaign,” *International Organization*, vol. 59, no. 2, pp. 419–449, 2005, [Online]. Available: <http://www.jstor.org/stable/3877910>.

A. Taylor, T. Alfvén, D. Hougendobler, and K. Buse, “Nonbinding Legal Instruments in Governance for Global Health: Lessons from the Global AIDS Reporting Mechanism,” *Journal of Law, Medicine & Ethics*, vol. 42, no. 1, pp. 72–87, Spring 2014, [Online]. Available: <http://search.ebscohost.com/login.aspx?direct=true&db=rzh&AN=103958714&site=ehost-live&scope=site>.

J. Taylor, “Crises in civil society organisations: opportunities for transformation,” *Development in Practice*, vol. 26, no. 5, pp. 663–669, Jul. 2016, doi: [10.1080/09614524.2016.1189878](https://doi.org/10.1080/09614524.2016.1189878).

C. Triantaphyllis, T. Ezer, and J. Cohen, “Twenty Mechanisms for Addressing Torture in Healthcare,” Open Society Foundations, Aug. 2012. [Online]. Available: <https://www.opensocietyfoundations.org/sites/default/files/twenty-mechanisms-addressing-torture-health-care-20120829.pdf>.

I. Varentsov, “As funding from the Global Fund is phased out, Bulgaria struggles to find sustainable financing,” *AIDSPAN*, Sep. 19, 2017. <http://www.aidspan.org/gfo_article/funding-global-fund-phased-out-bulgaria-struggles-find-sustainable-financing>.

J.-A. Walker, “Civil Society, the Challenge to the Authoritarian State, and the Consolidation of Democracy in Nigeria,” *Issue: A Journal of Opinion*, vol. 27, no. 1, pp. 54–58, 1999, doi: [10.2307/1167007](https://doi.org/10.2307/1167007).

K. West Slevin and C. Green, “Accountability and Transparency for Public Health Policy: Advancing Country Ownership,” Health Policy Project, Washington, DC, Apr. 2013. [Online]. Available: <http://www.healthpolicyproject.com/pubs/194_GovernenceBrief.pdf>.

K. West Slevin and C. Green, “Networking and Coalition Building for Health Advocacy: Advancing Country Ownership,” Health Policy Project, Apr. 2013. [Online]. Available: <http://www.healthpolicyproject.com/pubs/195_NetworksBrief.pdf>.

T. Windau-Melmer, “A Guide for Advocating for Respectful Maternity Care,” Futures Group, Health Policy Project, Washington, DC. [Online]. Available: <http://www.healthpolicyproject.com/pubs/189_RMCGuideFINAL.pdf>.

T. Zardiashvili, “New website in EECA provides information in Russian related to Global Fund policies, funded programs, etc.,” *AIDSPAN*, Sep. 04, 2016. <http://www.aidspan.org/gfo_article/new-website-eeca-provides-information-russian-related-global-fund-policies-funded>.

T. Zardiashvili, “New ARV database being piloted in the EECA region,” *AIDSPAN*, Apr. 18, 2017. <http://www.aidspan.org/gfo_article/new-arv-database-being-piloted-eeca-region>.

T. Zardiashvili, “How the Global Fund’s policy on CCM composition helped to boost LGBT participation in decision-making in the EECA,” *AIDSPAN*, Jun. 27, 2017. <http://www.aidspan.org/gfo_article/how-global-fund%E2%80%99s-policy-ccm-composition-helped-boost-lgbt-participation-decision-making>.

“Background paper: Civil society challenges,” INTRAC, Oxford, UK, 2008.

“Background paper: The challenge - getting civil society back on track,” INTRAC, Oxford, UK, 2008.

“INTRAC Review of Activities 2011-13,” INTRAC, Oxford, UK, Sep. 2011. [Online]. Available: <https://www.intrac.org/resources/intrac-annual-review-2011-13/>.

“Partnering with Civil Society: 12 Lessons from DAC Peer Reviews,” OECD, Paris, France, 2012. [Online]. Available: <http://www.oecd.org/dac/peer-reviews/12%20Lessons%20Partnering%20with%20Civil%20Society.pdf>.

“Policy Activities and Accomplishments - 2012,” Health Policy Project, 2012. [Online]. Available: <http://www.healthpolicyproject.com/ns/docs/Policy_Activities_2012.pdf>.

“Supporting Partners to Develop their Capacity: 12 Lessons from DAC Peer Reviews,” OECD, Paris, France, 2012. [Online]. Available: <http://www.oecd.org/dac/peer-reviews/12lessonscapdev.pdf>.

“HPP Governance Activities and Accomplishments - 2013,” 2013. [Online]. Available: <http://www.healthpolicyproject.com/ns/docs/governance_accomplishments_2013.pdf>.

“Health Policy Project and the Global Forum on MSM & HIV unite efforts for a focused response to HIV among key populations throughout the world,” May 01, 2013. <http://www.healthpolicyproject.com/index.cfm?id=MSMConsortium>.

“Study on Support to Civil Society through Multi-Donor Funds: Final Report,” INTRAC, Jan. 2014. [Online]. Available: <https://www.intrac.org/wpcms/wp-content/uploads/2016/09/Study_on_Support_to_Civil_Society_through_Multi-Donor_Funds.pdf>.

“Building sustainability of civil society: Beyond resourcing,” INTRAC, Oxford, UK, Nov. 2014. [Online]. Available: <https://www.intrac.org/wpcms/wp-content/uploads/2016/09/Building-sustainability-of-civil-society-Beyond-resourcing.-Reflections-from-INTRAC-staff-and-associates.pdf>.

“Strengthening Women’s Capacity to Influence Health Policy Making: How Coaching Helps Translate Learning into Action,” Health Policy Project, Washington, DC, Dec. 2014. [Online]. Available: <http://www.healthpolicyproject.com/pubs/268_WomensLeadershipBriefFINAL.pdf>.

“Readiness Assessment: Moving Towards a Country-Led and Country-Financed HIV Response for Key Populations,” Health Policy Project, 2015. [Online]. Available: <http://www.healthpolicyproject.com/pubs/462_SIDChecklistfillableinreader.pdf>.

“Amplifying Voices: Decade Edition 2005-2015,” Open Society Initiative for Eastern Africa, Nairobi, Kenya, 2016. [Online]. Available: <https://www.opensocietyfoundations.org/sites/default/files/amplifying-voices-decade-edition-20161012.pdf>.

“Botswana - How the Decline in PEPFAR Funding Could Affect Key Populations,” Brief, Mar. 2016. [Online]. Available: <http://www.healthpolicyproject.com/pubs/462_HPPBotswanaBriefMarchFINAL.PDF>.

“Clearing Up Some Myths About Sex Work,” *Open Society Foundations*. <https://www.opensocietyfoundations.org/explainers/understanding-sex-work-open-society>.

“Involving the Community in Responding to TB/HIV: Outcomes of Community-Led Monitoring and Advocacy,” Open Society Foundations. [Online]. Available: <https://www.opensocietyfoundations.org/sites/default/files/TBHIV_Community_Involvement.pdf>.

“Open Society 2010-2011,” Open Society Foundations, Annual Report. [Online]. Available: <https://www.opensocietyfoundations.org/sites/default/files/open-society-20120515.pdf>.

“Software, Models, and Tools.” <http://www.healthpolicyproject.com/index.cfm?id=topics-SoftwareModelsTools>.
